# Improved Processing Speed Is a Potential Mechanism by Which Exercise Reduces Falls

**DOI:** 10.1093/geroni/igaa057.759

**Published:** 2020-12-16

**Authors:** Teresa Liu-Ambrose, Jennifer Davis, Ryan Falck, Karim Khan

**Affiliations:** 1 University of British Columbia, Vancouver, British Columbia, Canada; 2 University of British Columbia, Kelowna, British Columbia, Canada

## Abstract

A 12-month trial demonstrated the Otago Exercise Program (OEP), a home-based exercise program of strength and balance retraining exercises, significantly reduced the rate of subsequent falls among 344 older adults receiving care after a fall (JAMA, 2019). A significant improvement in processing speed, as measured by the Digit Symbol Substitute Test (DSST), was also observed. Given the DSST is a predictor of falls, we conducted mediation analyses to determine whether improved DSST mediated the effects of OEP on rate of: 1) total falls; 2) non-injurious falls; 3) mild injurious falls; and 4) severe injurious falls over the 12-month trial. Our causal mediation analyses were conducted using the mediation package in R, using quasi-Bayesian estimates and 95% confidence intervals. Compared with usual care, OEP significantly reduced the rate of total falls (IRR= 0.64; 95% CI: 0.44, 0.91; p= 0.013) and mild injurious falls (IRR= 0.49; 95% CI: 0.31, 0.77; p= 0.002). Improved DSST score was also associated with lower mild injurious fall rates (IRR= 0.95; 95% CI: [0.91, 0.99]; p= 0.014). Formal mediation analyses showed that improved DSST was a significant mediator of the effect of OEP on the rate of mild injurious falls (95% CI: -0.15, 0.00; p= 0.036). Improved processing speed may be a mechanism by which exercise reduces mild injurious falls.

